# Traditional Visual Search vs. X-Ray Image Inspection in Students and Professionals: Are the Same Visual-Cognitive Abilities Needed?

**DOI:** 10.3389/fpsyg.2019.00525

**Published:** 2019-03-21

**Authors:** Nicole Hättenschwiler, Sarah Merks, Yanik Sterchi, Adrian Schwaninger

**Affiliations:** School of Applied Psychology, University of Applied Sciences and Arts Northwestern Switzerland, Olten, Switzerland

**Keywords:** visual search, visual inspection, letter search task, X-ray image inspection, visual-cognitive abilities, students, professionals

## Abstract

The act of looking for targets amongst an array of distractors is a cognitive task that has been studied extensively over many decades and has many real-world applications. Research shows that specific visual-cognitive abilities are needed to efficiently and effectively locate a target among distractors. It is, however, not always clear whether the results from traditional, simplified visual search tasks conducted by students will extrapolate to an applied inspection tasks in which professionals search for targets that are more complex, ambiguous, and less salient. More concretely, there are several potential challenges when interpreting traditional visual search results in terms of their implications for the X-ray image inspection task. In this study, we tested whether a theoretical intelligence model with known facets of visual-cognitive abilities (visual processing *Gv*, short-term memory *Gsm*, and processing speed *Gs*) can predict performance in both a traditional visual search task and an X-ray image inspection task in both students and professionals. Results showed that visual search ability as measured with a traditional visual search task is not comparable to an applied X-ray image inspection task. Even though both tasks require aspects of the same visual-cognitive abilities, the overlap between the tasks was small. We concluded that different aspects of visual-cognitive abilities predict performance on the measured tasks. Furthermore, although our tested populations were comparable in terms of performance predictors based on visual-cognitive abilities, professionals outperformed students on an applied X-ray image inspection task. Hence, inferences from our research questions have to be treated with caution, because the comparability of the two populations depends on the task.

## Introduction

Visual search, the act of looking for targets amongst an array of distractors, is a demanding cognitive task (e.g., Treisman and Gelade, 1980) that has many real-world applications. Some individuals conduct visual search tasks professionally, for example, airport security officers (screeners) who visually inspect X-ray images of passenger baggage to search for prohibited items or radiologists who are looking for cancer in mammograms. Because search errors can have huge, even fatal, consequences in such professional applications, research can provide a valuable contribution by reducing these errors. The ability to locate a target amongst an array of distractors has been studied extensively over many decades (for reviews see e.g., Carrasco, 2011, 2014, 2018; Eckstein, 2011; Nakayama and Martini, 2011; Humphreys and Mavritsaki, 2012; Chan and Hayward, 2013). Research also shows that specific visual-cognitive abilities are needed to effectively and efficiently locate a target among distractors. However, many of the studies on visual search have been conducted using traditional, simplified tasks with salient stimuli and have been done with non-professional searchers (mostly students). These studies have provided vital insights into the cognitive mechanisms underlying visual search due to the high experimental control. It is, however, not clear whether the results from such traditional, simplified visual search tasks extrapolate to real-world inspection tasks in which professionals search for targets that are more complex, ambiguous, and/or less salient (e.g., Biggs and Mitroff, 2014; Radvansky and Ashcraft, 2016, p. 257). It is also unclear to what extent findings based on student samples can be transferred to professionals who often rely on extensive training and experience. To address these issues, we first introduce visual search in general before comparing insights on traditional visual search tasks vs. a real-world application, namely X-ray image inspection, and considering the populations conducting these search tasks.

### Visual Search and Visual Search Tasks

Visual search typically involves an active scan of the visual environment for a particular target among many distractors. This is a demanding cognitive task requiring specific visual-cognitive abilities (Treisman and Gelade, 1980). Over the past several decades, psychological research has made tremendous headway in understanding the underlying cognitive processes when performing visual search tasks and the mechanisms that allow a successful identification of target items (Clark et al., 2012). Search thereby involves several processes such as perception (i.e., processing and interpreting visual features), attention (i.e., allocating resources to the relevant areas of a visual area), and memory (for reviews see e.g., Carrasco, 2011, 2014, 2018; Eckstein, 2011; Nakayama and Martini, 2011; Humphreys and Mavritsaki, 2012; Chan and Hayward, 2013; storing a representation of the target item or items). To conduct visual search and inspection, certain visual-cognitive abilities such as attention, memory, visual processing, or processing speed have been found to correlate with higher performance.

A known example of a traditional visual search task that has been studied in many variations is the *L/T-letter search task*. According to Treisman and Gelade (1980), this is called a conjunction search task. Conjunction search involves distractors (or a group of distractors) that may differ from each other but exhibit at least one common feature with the target and therefore require a combination of features to distinguish them (Shen et al., 2003). For example, the letters T and L share exactly the same features, differing only in their spatial arrangement (*L/T-letter* search task: Treisman and Gelade, 1980). In one variation of this task, participants are asked to identify the perfectly shaped letter *T* (target) surrounded by many distractor letters including *Ls* and symmetrical and asymmetrical *Ts*. The efficiency of such a conjunction search in terms of accuracy and reaction time depends on the distractor ratio and the number of distractors present (McElree and Carrasco, 1999), and the negative effect of limiting reaction time on accuracy is alleviated by training (Reavis et al., 2016).

In more complex real-world visual search applications, humans sometimes conduct visual search and inspection tasks professionally. For example, radiologists inspect mammograms for cancer (e.g., Nodine and Kundel, 1987; Krupinski, 1996; Horowitz, 2017) or screeners inspect X-ray images for prohibited items (Drury, 1975; Koller et al., 2009; Wales et al., 2009; Mitroff et al., 2015). In these scenarios, professionals search for targets that are less artificial and more familiar to them. They must use their prior knowledge in order to accurately and efficiently locate more ambiguous targets (Wolfe et al., 2019) such as guns and knives or cancer cells and so forth among distractors with much more complex features compared to a traditional conjunction search task. Searching for familiar stimuli relies on object recognition (Wolfe, 1998). Here, top-down processing allows searchers to more efficiently identify targets with greater complexity (Zhaoping and Frith, 2011). X-ray image inspection is therefore best described as a search and decision task (Spitz and Drury, 1978; Koller et al., 2009) that relies more heavily on the decision component compared to traditional search tasks with unambiguous stimuli. Nonetheless, visual search with complex objects is assumed to rely on the same active scanning processes as conjunction search (e.g., L/T-letter search task) with less complex, contrived laboratory stimuli (Alexander and Zelinsky, 2011, 2012).

When translating results from a traditional visual search task such as an L/T-letter search task to X-ray image inspection and vice versa, it is necessary to consider differences in the nature of stimuli and the characteristics of searchers. Differences in stimuli include target and distractor complexity as well as the requirement of domain-specific knowledge of the searcher in order to successfully recognize the target (e.g., Biggs and Mitroff, 2014). On the other hand, targets in a traditional visual search task are often commonly known to have salient shapes and colors, whereas targets in X-ray image inspection tasks are not well-specified, not salient, and not predictable through the context (Bravo and Farid, 2004). The large variety of potential threat items and distracting objects in passenger bags makes X-ray image inspection a difficult task (Hättenschwiler et al., 2015; Sterchi et al., 2017). This calls for domain-specific knowledge, because screeners must know which items are prohibited and what they look like in X-ray images (Schwaninger, 2004, 2005, 2006). Due to the differences between traditional visual search tasks and X-ray image inspection, it is unclear whether they require the same visual-cognitive abilities. We shall discuss this in the next section. Because research on traditional visual search tasks and X-ray image inspection differs in regard to not only the task but also the examined population, we shall discuss differences between students and professional screeners in section Populations Conducting Visual Search.

### Cognitive Abilities for Visual Search

Both traditional visual search and X-ray image inspection can be characterized as a basic, core cognitive task. As defined by Carroll (1993), a cognitive task is any task in which correct processing of mental information is critical for successful performance. Therefore, specific cognitive abilities are needed to perform such a task successfully. These abilities can be assessed with specific correlated measures that can predict performance. With regard to visual search and inspection, certain visual-cognitive abilities such as attention, memory, visual processing, or processing speed have been found to correlate with higher performance (Wolfe et al., 2002; Bolfing and Schwaninger, 2009). If individual differences in performance are found on visual search or inspection tasks, these can be seen as the direct manifestation of differences in an underlying ability or latent trait (Carroll, 1993, 2003).

There is a large number of such abilities and many theories aiming to integrate cognitive abilities. Today, the Cattell–Horn–Carroll theory (CHC) is widely accepted as the most comprehensive and empirically supported theory on the structure of human cognitive abilities, and it informs a substantial body of research and the ongoing development of intelligence tests (McGrew, 2005). The CHC theory states that the relationships among these cognitive abilities can be derived by classifying them into three different strata: Stratum I, “narrow” abilities; Stratum II, “broad abilities”; and Stratum III, a single general ability also called *g* (Flanagan and Harrison, 2005). The factors describe stable and observable differences between individuals. However, the structure of the three strata is hierarchical, meaning that the abilities within one stratum (e.g., the narrow abilities of Stratum I) are positively intercorrelated, thereby allowing an estimation of Stratum II, the broad abilities. Likewise, the abilities of Stratum II have non-zero intercorrelations, thereby allowing an estimation of Stratum III. Hence, whereas the abilities within Strata I or II are related, a large amount of evidence shows that they are unique and reliably distinguishable (see e.g., Keith and Reynolds, 2012).

Visual processing (*Gv*), short-term memory (*Gsm*), and processing speed (*Gs*) are broad Stratum II abilities that are accepted components with a known influence on visual search and inspection performance. Therefore, they are included in most commonly used measures of intelligence (e.g., Stanford-Binet: Roid, 2003a,b; Wechsler Intelligence Scale: Wechsler, 1997). Visual processing (*Gv*) describes a broad ability to perceive, analyze, synthesize, and think in visual patterns, including the ability to store and recall visual representations. Short-term memory (*Gsm*) is characterized as the ability to apprehend and hold information in immediate awareness and then perform a set of cognitive operations on this information within a few seconds. Because analyzing, synthesizing, and thinking in visual patterns are also cognitive operations, *Gv* and *Gsm* are closely related, but can be distinguished by the limited capacity of short-term memory. Processing speed (*Gs*) describes the ability to quickly and accurately perceive visual details, similarities, and differences.

Several studies have confirmed the influence of higher scores in *Gv, Gsm*, and *Gs* on better performance in traditional visual search tasks (Eriksen and Schultz, 1979; Alvarez and Cavanagh, 2004). Cognitive abilities have also been linked to inspection performance in studies on X-ray image inspection with professionals (e.g., Schwaninger et al., 2004; Hardmeier et al., 2005; Hardmeier and Schwaninger, 2008). Detection performance decreases significantly if threat items are shown in close-packed bags, if threats are more superimposed by other items, and if they are shown in an unusual view. Studies linked the influence of mental rotation and figure–ground segregation, which are narrow abilities of visual processing (*Gv*), to higher X-ray image inspection performance (Wolfe et al., 2002; Bolfing and Schwaninger, 2009). Items presented from unusual or rotated viewpoints become more difficult to detect (effect of viewpoint; Palmer et al., 1981). Similarly, the position of a prohibited item in a bag and its superposition by other objects (effect of superposition), or the number and types of items in a bag that could attract attention (effect of bag complexity) also affect the difficulty in recognizing prohibited items. Bag complexity comprises the factors clutter (disarrangement, textural noise, chaos, etc.) and opacity (X-ray penetration of objects; see Schwaninger et al., 2008). Memory capacity, which can be classified as short-term memory (*Gsm*), is strongly associated with visual inspection in general (e.g., Lavie and DeFockert, 2005; Poole and Kane, 2009; Roper et al., 2013). In addition, processing speed (*Gs*) might be relevant for the efficiency of the visual inspection task (Salthouse, 1996). Based on the reviewed literature, the question arises whether the same visual-cognitive abilities can predict performance in a traditional visual search task and an X-ray image inspection task.

### Populations Conducting Visual Search

As a positive correlation was found between certain visual-cognitive abilities and performance in X-ray screening, many European airports conduct preemployment assessments that test for these visual abilities and aptitudes when recruiting new personnel (e.g., X-Ray Object Recognition Test; see Hardmeier et al., 2005; Hardmeier and Schwaninger, 2008). Professional screeners conducting X-ray image inspection have therefore been selected accordingly, and they usually have a lot of experience on this specific task through many hours of training and years of job experience. In comparison, university students are the first choice as participants for traditional visual search research because they are an easily accessible population. Therefore, differences between professional screeners and students could be due either to characteristics of the searchers as a result of self and pre-employment selection or to training and job experience as professionals (Clark et al., 2012).

Training for threat detection has the goal of creating internal visual representations of objects and storing them in memory. To identify whether an object in an X-ray image is a threat or not, a searcher must successfully match the visual information of this object to representations stored in visual memory (Kosslyn, 1975, 1980). Depending on the similarity of objects and its features presented in an X-ray image to those stored in visual memory, the screener will then decide whether the respective object is harmless or not. More familiar objects therefore need fewer recognized features in order to be identified successfully (Koller et al., 2009). Detection of objects—known and especially unknown—should therefore improve with training because features become familiar and are recognized better through repeated exposure. For example, features of guns and knives are known from everyday life and can therefore also be detected by novices without specific experience or training. However, screeners have been exposed to these objects more often and have therefore more detailed and specific target templates and are more familiar with them (Koller et al., 2009). However, other prohibited items that are rather uncommon or have never been seen before (e.g., improvised explosive devices, IEDs) become very difficult to recognize for novices if they have not been trained to recognize certain features of these threats (Schwaninger, 2004, 2005).

### Current Study

Over the past several decades, psychological research has made tremendous headway in understanding the underlying cognitive processes when performing visual search tasks and the mechanisms that allow for the successful identification of target items (Clark et al., 2012).

However, most of the research on this theoretical basis was conducted with students using tasks applying artificial stimuli to allow for maximum experimental control (for reviews, see e.g., Duncan and Humphreys, 1989; Wolfe, 1994, 1998; Eckstein, 2011). It is therefore unclear to what extent professional X-ray image inspection relies on the same cognitive processes. Because the tasks in traditional visual search and X-ray image inspection are often conducted by different populations, it is also necessary to ask whether the two populations rely on the same cognitive processes. To date, no study has examined the influence of visual-cognitive abilities on visual search performance by comparing a traditional visual search task and an X-ray image inspection task.

Based on the literature on visual-cognitive abilities, we postulate a theoretical model in which several known facets (visual processing *Gv*, short-term memory *Gsm*, and processing speed *Gs*) can predict performance in a traditional visual search task and an X-ray image inspection task. We shall test this model on two populations (students and professionals) using the same experimental stimuli. This will provide an indication on whether the two populations require the same visual-cognitive abilities or whether visual-cognitive abilities can be compensated by experience and training in X-ray image inspection. To have a fair comparison, we created a traditional visual search task with *Ls* and *Ts* on a high difficulty level and an X-ray image inspection task with no need for domain-specific knowledge that included only black and white images as well as familiar target items such as guns and knives. Features of guns and knives as well as letters such as L or T, are known from everyday life experience and can therefore be recognized without specific experience and training. We used this comparison to address the following research questions: (1) Do different visual-cognitive abilities predict performance in a traditional visual search task and an X-ray image inspection task? (2) Do the results differ between students and professionals? Answers to these questions could provide important information on how well studies conducted with students and traditional visual search tasks can be generalized to professional X-ray image inspection.

## Methods

### Participants

Table 1 reports the participants' descriptives. 128 participants were *students* from the University of Applied Sciences and Arts Northwestern Switzerland. 112 participants were *professionals* (airport security screeners employed at an international airport) who were selected, qualified, trained, and certified according to the standards set by the appropriate national authority (civil aviation administration) in compliance with the relevant EU regulation (European Commission, 2015). The current research complied with the American Psychological Association Code of Ethics and was approved by the Institutional Review Board of the University of Applied Sciences and Arts Northwestern Switzerland.

**Table 1:** Description of participants.

	***N***	**Age**	**Gender**	**SPM**
Students	128	*M* = 25.7	74% female	*M* = 30.8
		*SD* = 6.4		*SD* = 3.0
Professionals	112	*M* = 43.7	55% female	*M* = 28.3
		*SD* = 11.9		*SD* = 4.2

*255 participants gave informed consent to be part of this experiment. 15 participants had to be excluded from statistical analyses (5.9% of the sample) due to a malfunction of a simulator (n = 4) or performance below chance (n = 11). Therefore, the final sample included 240 participants. SPM, Standard Progressive Matrices raw scores as a baseline measure of fluid intelligence*.

### Apparatus

We used six HP ProBooks 4730s and 4720s with Intel Core i5 2410M and 520M processors and 19″ TFT monitors. The six testing stations were separated, and the room was dimly lit for testing. Participants sat approximately 50 cm away from the monitor. Non-professional searchers were tested in the laboratory at the University of Applied Sciences and Arts. Professional searchers were tested at the test facilities of the Center for Adaptive Security Research and Applications (CASRA) using the same computers and monitors.

### Stimuli

#### Visual Cognitive Test Battery

A visual-cognitive test battery (VCTB) was developed to measure a broad spectrum of visual-cognitive abilities assessing a wide variety of narrow abilities underlying *visual processing* (*Gv*), *short-term memory* (*Gsm*), and *processing speed* (*Gs*) in order to make predictions on visual search performance. The VCTB consists of 10 standardized tests scales taken mostly from well-established intelligence tests based on the CHC theory of intelligence (Cattell, 1941; Horn, 1965; Carroll, 1993, 2003). Four scales came from a major German intelligence test, the Leistungsprüfsystem 2 (LPS-2; Kreuzpointner et al., 2013). Three tests were taken from a cognitive development test, that assesses visual perceptual weaknesses and strengths—the Test of Visual Perceptual Skills (TVPS-3; Martin, 2006). Another three scales were used from a Swiss online assessment test for students (WSI; Hell et al., 2009; Päßler and Hell, 2012) In addition, we included Raven's standardized progressive matrices (SPM; Horn, 2009) as a general measure of fluid intelligence. Because most scales were originally in paper-and-pencil format, we created computer-based versions. Table 2 reports the psychometrical criteria of the test scales.

**Table 2:** Psychometric criteria of the VCTB test scales (objectivity, reliability, validity).

**Test**	**Scale**	**Objectivity**	**Reliability**	**Validity**
LPS	LPS 6: Mental rotation (Gs) LPS 7: Number of surfaces (Gs) LPS 8: Shape Comparison (Gs) LPS 10: Row comparison (Gs)	Standardized	Cronbach's α: 0.86–0.94 Split-half: 0.81–0.96	Factor analyses Correlations with g
WSI	WSI Slices (Gsm) WSI Mental rotation (Gsm) WSI Unfold (Gsm)	–	–	–
TVPS	TVPS Visual Memory (Gv) TVPS Form Constancy (Gv) TVPS Figure Ground (Gv)	Standardized	Cronbach's α: 0.74 Test–Retest: 0.71	–
SPM	SPM: Speed-Test	Standardized	Cronbach's α: 0.97–1.00 Split-half: > 0.90 Test–Retest: 0.80–0.90	Correlations with nonverbal IQ

*Psychometric criteria are retrieved as follows: LPS from Kreuzpointner et al. (2013); TVPS from Brown et al. (2010); SPM from Horn (2009)*.

##### Visual processing (Gv)

**Figure 1:**
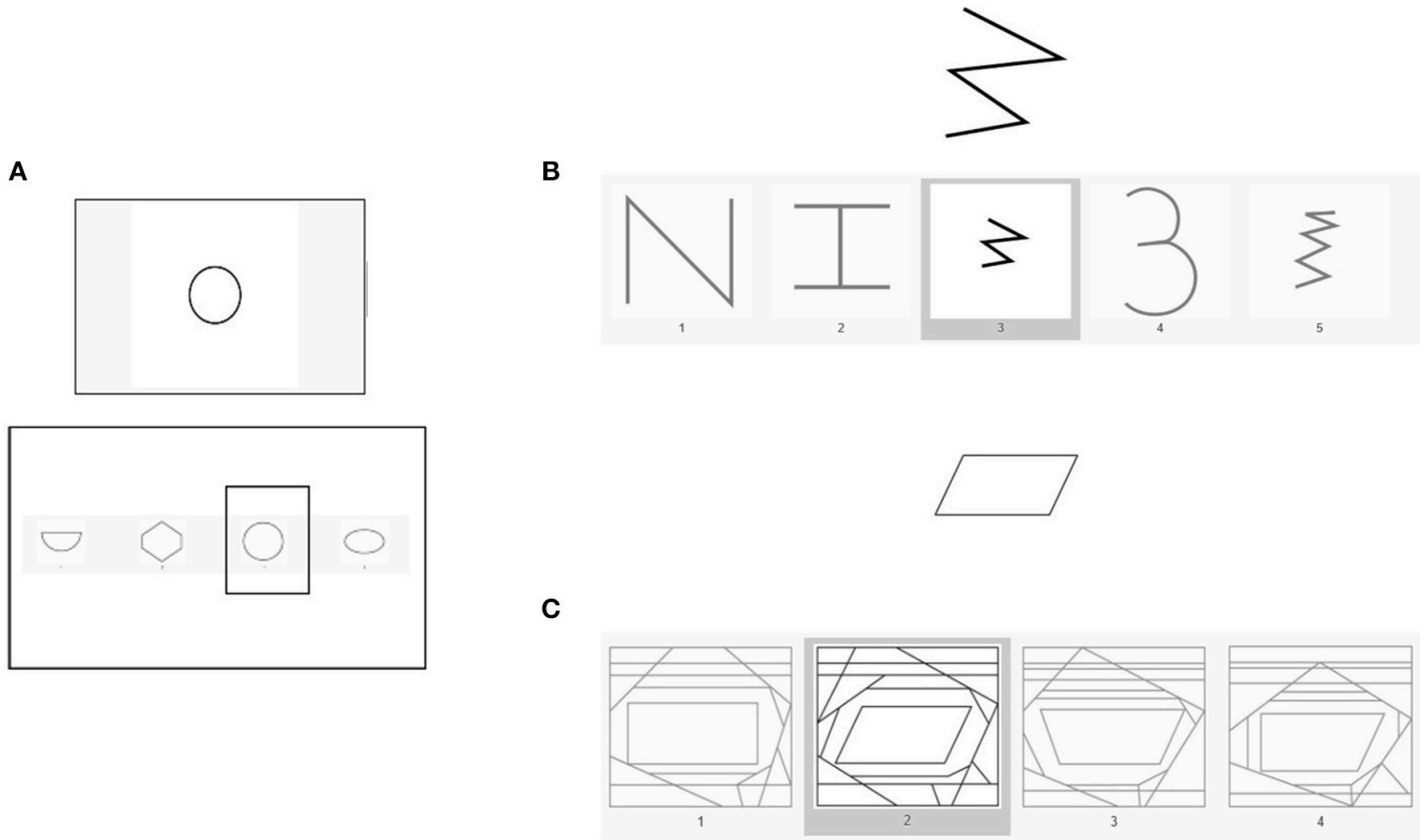
Image example of the three scales of TVPS-3: **(A)** visual memory, **(B)** form constancy, and **(C)** figure–ground segregation.

We assessed visual processing with three scales from the TVPS-3 (visual memory, form constancy and figure-ground segregation; see Figure 1). For visual memory, participants have to memorize a design for 5 s and then recognize this pattern from four alternatives presented on the next slide. The scale consists of 16 tasks and the score is the sum of correct responses. To measure form constancy, participants are instructed to find a target shape within five alternative, more complex patterns that can be rotated, increased, or decreased in size. There are 16 trials and the score is the number of correct responses. Figure-ground segregation is defined as the ability to recognize a target shape within a very cluttered, busy background. Participants have to choose one out of four complex patterns that include the target shape. There are 16 trials, and the score is the number of correct responses.

##### Short-term memory (Gsm)

**Figure 2:**
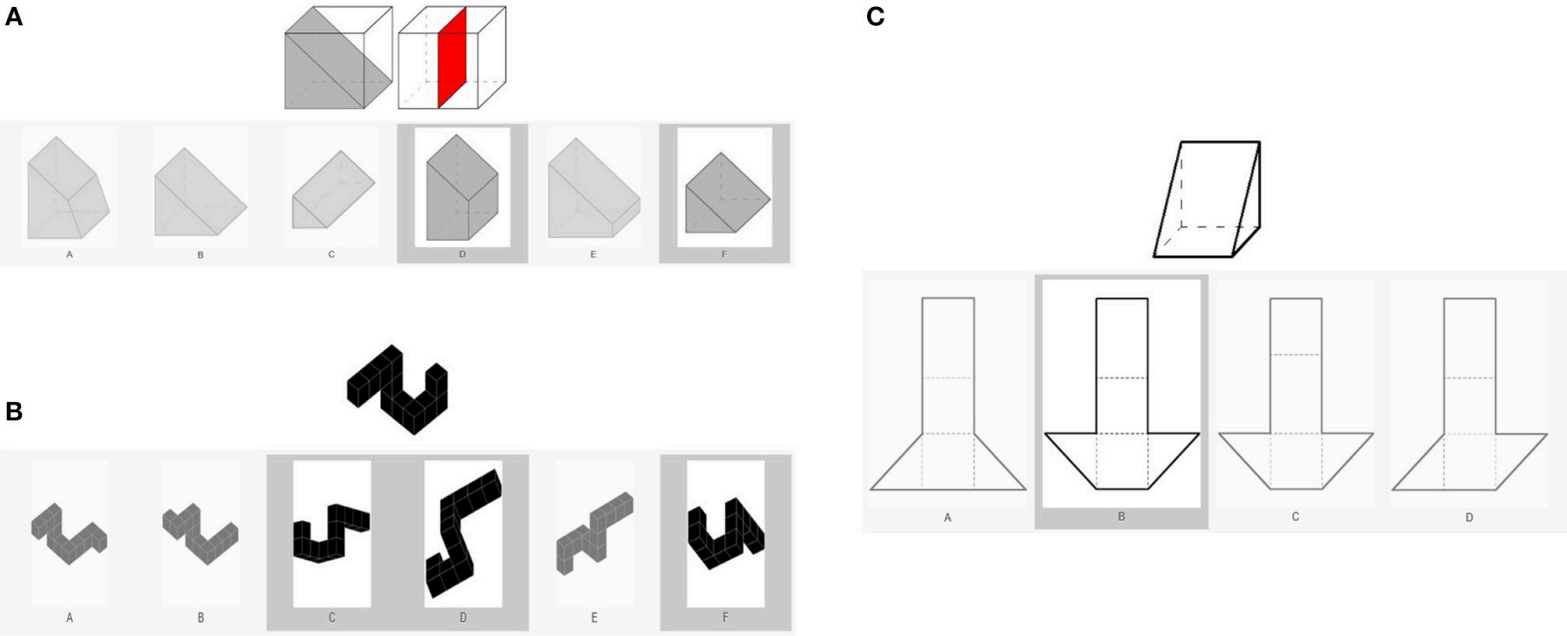
Image example of the three scales from the WSI: **(A)** slicing, **(B)** spatial rotation, and **(C)** unfold.

Short-term memory was measured using three scales from the WSI (slicing, spatial rotation, and unfold; Figure 2). Slicing can be referred to as another form of three-dimensional visualization. During the task, participants see a full three-dimensional object and next to this a cube with two or three dividers. The task is to visualize how the presented dividers slice the full objects and then choose all these pieces from a series of alternatives. Each correctly chosen piece is scored. We used spatial rotation to have another measure of the ability to mentally rotate objects. Participants see different three-dimensional objects. Besides one original figure, six additional figures are shown and the participant's task is to choose which of the figures represents the original figure when rotated or moved. The score is the number of correct responses. Unfold is another measure of visualization in which participants see a three-dimensional object and a series of folding templates. They then have to visualize the template that forms the original three-dimensional object. The score is the number of correct responses.

##### Processing speed (Gs)

**Figure 3:**
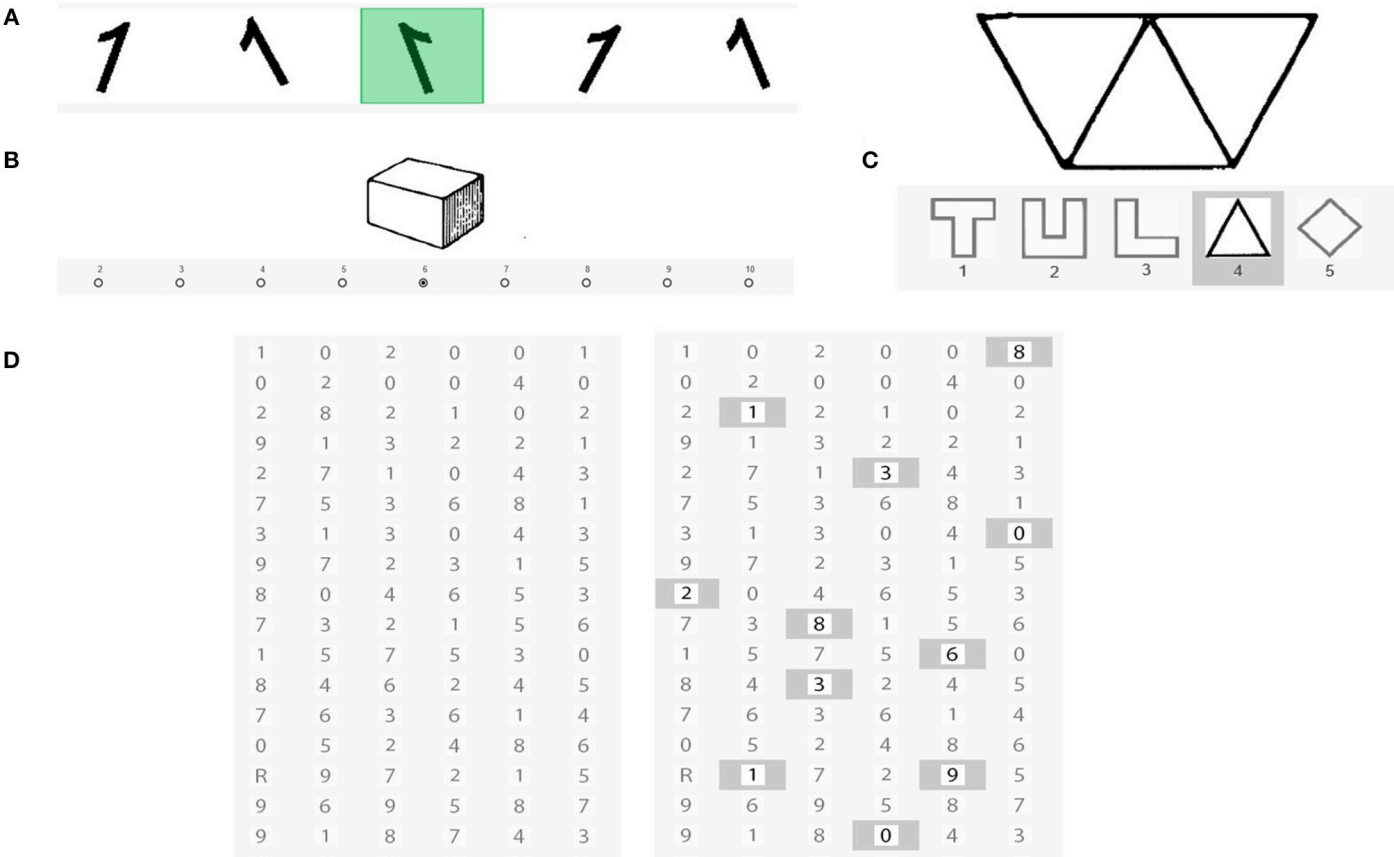
Image example of Subtests 6, 7, 8, and 10 of the LPS-2: **(A)** spatial relation, **(B)** visualization, **(C)** perceptual speed, and **(D)** scan/search.

Processing speed was measured with Subtests 6, 7, 8, and 10 of the LPS-2 (spatial relation, visualization, perceptual speed, and scan/search; see Figure 3). All scales measure the ability to quickly and accurately perceive visual details, similarities, and differences. Spatial relation was measured with Subtest 6 in which participants have to search for the one mirror-inverted number or letter in a list. Several signs can be rotated, but only one sign is mirrored and has to be marked. The scale consists of 40 trials. Scored are the correct responses reached within 2 min. We measured visualization, the ability to visualize a three-dimensional object, with Subtest 7. The participants' task is to determine the number of surfaces of a given geometrical figure. To do this, they need to visualize the figure in a three-dimensional space by counting the number of sides of the given object and indicating the number of sides by clicking on the corresponding number. There are 40 trials. The score is determined by counting the number of correct responses reached within 3 min. In subtest 8, perceptual speed, the participants' task is to recognize one out of five shapes embedded in a more complex pattern. The scale contains 40 patterns of increasing complexity. The score is the number of correct responses reached within 2 min. In subtest 10, scan and search, participants have to compare two lists of characters shown next to each other and mark characters that are different in the second list. Whereas, some rows are identical, others can differ in more than one character. The score is the number of correct markings within 2 min.

##### Fluid intelligence

The Raven Standard Progressive Matrices Plus (SPM) is a language-independent test of fluid intelligence. Participants see a matrix of logical patterns and have to choose the missing piece out of six to eight abstract figures (Raven et al., 2003). The tests consists of 48 items of increasing complexity. The score is the number of correct responses reached within 10 min.

#### Simulated Baggage Screening Task

The simulated baggage screening task (SBST) was created based on the X-Ray Object Recognition Test (X-Ray ORT, Schwaninger et al., 2005; Hardmeier et al., 2006). The original ORT was designed to measure how well professional and non-professional searchers can cope with image-based factors that impact on the detection of prohibited items (viewpoint, superposition, and bag complexity) rather than measuring knowledge-based determinants of threat detection performance (which is largely dependent on training). To this end, guns and knives are used in the ORT, that is, object shapes that can be assumed to be known by most people. All X-ray images are in black and white, because colors mainly diagnose the material of the objects in the bag, and thus, could primarily help experts. In addition, all guns and knives are shown for 10 s before the test starts, thereby further reducing the role of knowledge-based factors in this test.

**Figure 4:**
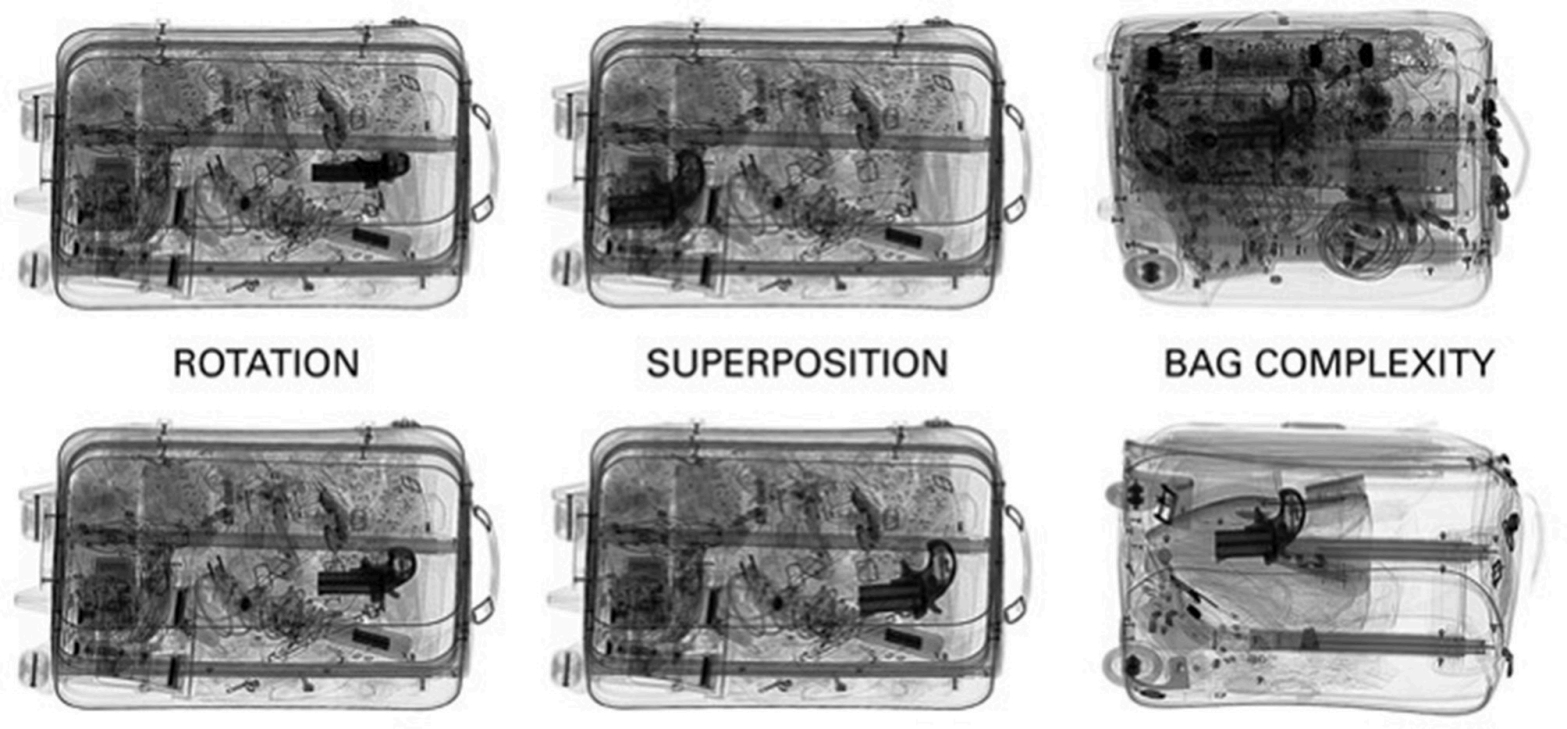
Examples of X-ray images varying in rotation of the threat object (viewpoint effect), superposition, and bag complexity.

The SBST created for this experiment included 256 X-ray images, with one half of the images containing threat item. As threats, eight guns and eight knives with common shapes were used. The X-ray images used in the SBST vary systematically in image difficulty by varying the degree of view difficulty, bag complexity, and superposition, both independently, and in combination (see Figure 4 for examples). Therefore, each gun and each knife was displayed in an easy view and a rotated view to measure the effect of viewpoint. Each view was combined with two bags of low complexity: once with low superposition, and once with high superposition. These combinations were also generated using two close-packed bags with a higher degree of bag complexity. In addition, each bag was presented once with and once without a threat item. Thus, there were a total of 256 trials: 2 weapons (guns, knives) × 8 (exemplars) × 2 (views) × 2 (bag complexities) × 2 (superpositions) × 2 (harmless vs. threat images). The test was divided into four blocks of 64 trials each. The order of blocks was counterbalanced across four groups of participants using a Latin square. Within each block, the order of trials was random.

#### L/T-Letter Search Task

**Figure 5:**
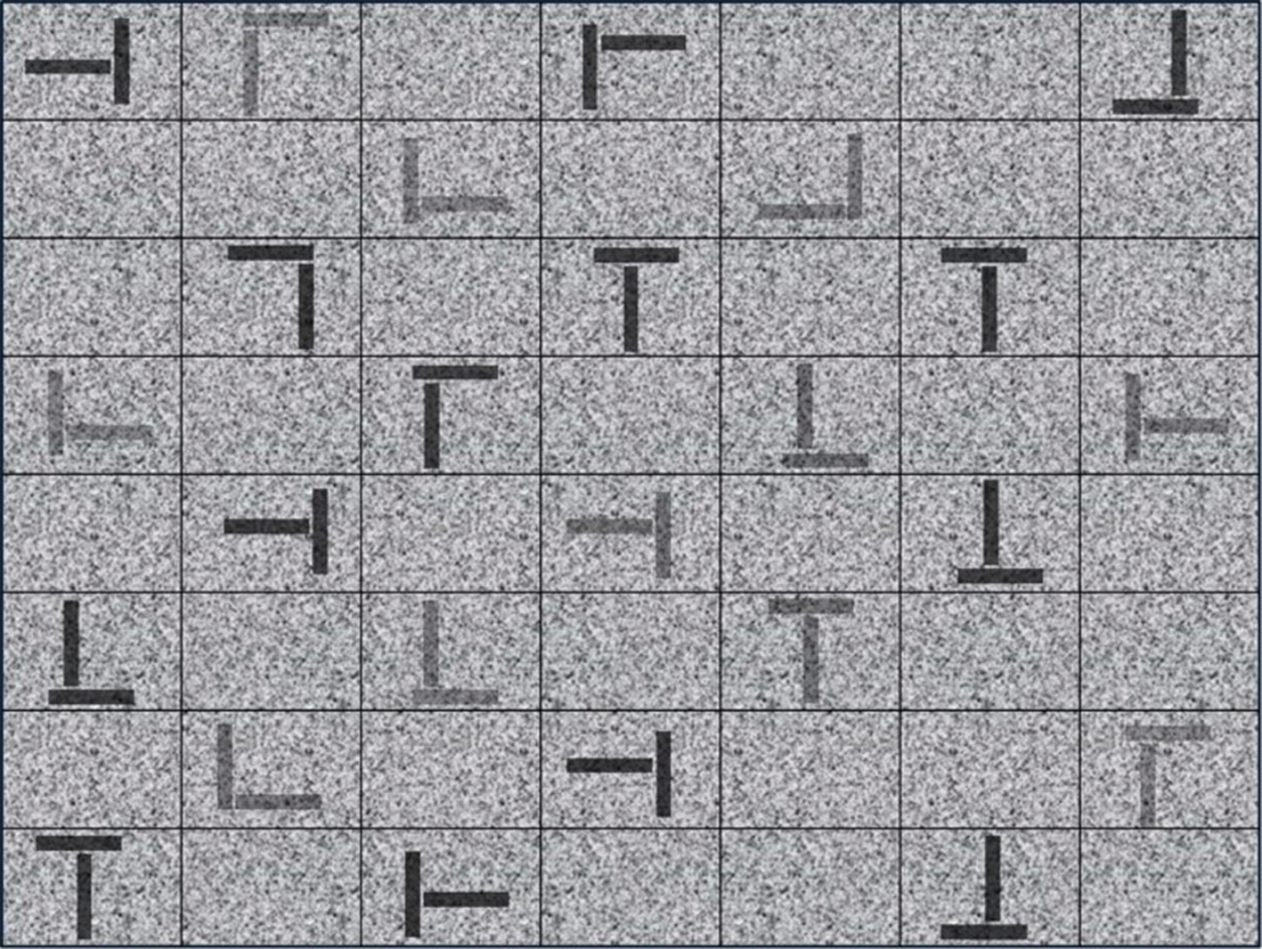
Example of an image from the L/T-letter search task. Image containing several pseudo-Ls as distractors and one target T against a gray background.

Comparable to previous research using laboratory visual search tasks, we created an L/T-letter search task to evaluate visual search abilities that are independent of a specific domain. In line with Biggs et al. (2013), we created a test with an increasing difficulty level and a search and decision component. The test consisted of 96 trials. Each image comprised 25 pseudo-Ls as distractors, and one-half of the images contained one target T against a gray background (see Figure 5 as an example). Items were randomly located in a 8 × 7 grid. Each item comprised two perpendicular black lines that varied on six levels of transparency (70, 67, 65, 40, 35, and 30%) and four levels of rotation. Target Ts had a crossbar directly in the middle, whereas distractor Ls had a crossbar sliding to variable distances away from the center. The distractor stimuli varied in shape with some being very similar to the target Ts. This increased task difficulty in line with a complex conjunction search task. All items were distractors for the target-absent condition, and in the target-present condition, all items were distractors except for one target T.

### Procedure

All participants were first tested with the visual-cognitive test battery (VCTB). In addition, the participants conducted a basic visual L/T- letter search task. In a second session, all participants were invited to conduct a simulated baggage screening task (SBST) using single-view X-ray images.

For the VCTB, all tests were computer-based and not conducted in the original paper-and-pencil format. Each of the 10 subtests started with general instructions followed by an example. The same procedure was applied to the SPM following the VCTB scales. The test was divided into three blocks and participants were asked to take a break of 10–15 min between blocks. For the SBST, participants came to the testing facilities again, approximately 2 weeks later. Each participant sat approximately 50 cm away from the monitor. The X-ray images covered about two-thirds of the screen. After task instructions, an introductory session followed using two guns and two knives not displayed in the test phase. In each trial, an X-ray image of a piece of luggage was presented for a maximum of 4 s. We chose this duration to match the demands of high passenger flow in which average X-ray image inspection time at checkpoints is in the range of 3–5 s. The participants' task was to decide as accurately and as quickly as possible whether the bag was OK (no threat item) or NOT OK (a gun or knife present) by clicking on the respective button. Prior to the actual test phase, the eight guns and eight knives used in the test were each presented for 10 s. Feedback was provided after each trial, but only in the introductory phase. For the L/T- letter search task, the same computers and monitors were used as for the SBST. Again, participants sat approximately 50 cm away from the monitor and the images covered about two-thirds of the screen. Each trial started with a fixation cross in the middle of the screen. After 0.5 s, a grid with 25 stimuli was presented for a maximum of 15 s. Each grid had 0 or 1 T's. If participants recognized a target T, they had to press “Y” on the keyboard and then mark the target T with the mouse. If they did not see a target T, they had to press “space” on the keyboard. As soon as participants marked the target T with the mouse or pressed the spacebar, the next trial started. If there was no decision after 15 s, the next trial started.

### Analyses

Both tasks used in this experiment can be described as a visual inspection consisting of visual search and decision (Spitz and Drury, 1978; Koller et al., 2009; Wales et al., 2009). The outcome of this task is based on the searchers decisions on whether a target is present or absent. According to signal detection theory (SDT) (Green and Swets, 1966), there are four possible outcomes depending on stimuli and participant responses (Table 3). Because individuals with identical detection ability can have different levels of hit rate and false alarm rate due to different response tendencies, it is often more appropriate to express detection performance in terms of a sensitivity measure (Green and Swets, 1966; Macmillan and Creelman, 2005). We therefore used *d*′ as detection measure for the L/T-letter search task based on the following formula in which *z* refers to the inverse of the cumulative distribution function of the standard normal distribution (Green and Swets, 1966; Macmillan and Creelman, 2005):

(1) $$\begin{array}{l} d^{\prime }=z\left(\text{HR}\right)-z\left(\text{FAR}\right) \end{array}$$

*d*′ is based on the equal variance Gaussian model, a common model of SDT (Pastore et al., 2003). SDT can also assume other underlying evidence distributions. One example is a SDT model that assumes the two evidence distributions to be normal but with unequal variance. For a given ratio *s* between the standard deviation of the target-present and target-absent distribution, the resulting *z*ROC has slope *s*. For this SDT model, Macmillan and Creelman (2005) propose using Simpson and Fitter's (1973) detection measure:

(2) $$\begin{array}{l} d_{a}=\;\sqrt{\frac{2}{1+s^{2}}}\times \left[z\left(HR\right)-sz\left(FAR\right)\right] \end{array}$$

Concerning the task of X-ray screening, several studies have raised doubts about the equal variance Gaussian model. Wolfe et al. (2007) proposes a *z*ROC slope of 0.6, which indicates that the noise (target-absent) distribution has a smaller standard deviation than the signal-plus-noise (target-present) distribution. Further publications (Van Wert et al., 2009; Godwin et al., 2010) have reported *z*ROC slopes similar to those reported by Wolfe et al. (2007) while a study reported by Wolfe and Van Wert (2010) found a slope of 0.56 and a study by Sterchi et al. (2019) a slope of 0.5 to fit the data more accurately. In our study, data from the basic visual search task (L/T-letter search task) were analyzed under the assumption of an equal variance model using *d*′, whereas data from the X-ray image inspection task SBST were analyzed under the assumption of an unequal variance model with a *z*ROC slope of 0.5 using *d*_*a*_^1^.

**Table 3:** Definition of hit, false alarm, miss, and correct rejection according to SDT (Green and Swets, 1966).

**Stimulus**	**Target-present response**	**Target-absent response**
**Target-present stimulus**	Hit	Miss
**Target-absent stimulus**	False alarm	Correct rejection

In a first step, we examined descriptive statistics (means and standard deviations) as well as correlations (Spearman correlations; Spearman, 1927) with basic functions of R Statistics version 3.4.4 (R Core Team, 2018). We then performed confirmatory factor analysis (CFA) using maximum likelihood methods of estimation with the package “lavaan” (Rosseel, 2012) in R Statistics version 3.4.4 (R Core Team, 2018). We report factor loadings of CFA, which should be minimally 0.50 and optimally higher than 0.70. To estimate the goodness of fit for the models, we report Chi^2^ values, the comparative fit index (CFI), the Tucker–Lewis index (TLI), and the root-mean-square error of approximation (RMSEA). CFI and TLI values close to 0.95 or higher (Hu and Bentler, 1999) and RMSEA values up to 0.07 (Steiger, 2007) indicate a good fit between the data and the proposed model. For the multiple regression analyses, predictors were entered into the regression using the “enter method” (forced entry). For results, we report *R*^2^, *F*, and *p* to evaluate the overall model fit. Furthermore, we report β, *SE, t*, and *p* for each predictor. In order to compare regression models, we used Wald's test and the Bayes factor. Bayes factor was calculated with the package “BayesFactor” (Morey et al., 2018) in R Statistics version 3.4.4 (R Core Team, 2018). The interpretation of the Bayes factor as evidence for the alternative hypothesis was reported in line with Raftery (1995).

## Results

We first report descriptive statistics and Spearman correlations. In accordance with the CHC model of intelligence (e.g., Flanagan and Dixon, 2013), we then computed a CFA over the VCTB scales with three latent factors: *visual processing* (*Gv*), *short-term memory* (*Gsm*), and *perceptual speed* (*Gs*) in order to confirm the construct validity of the used VCTB. Further, we performed multiple regression analyses to test whether the *z*-standardized summarized scale scores of *Gv*, *Gms*, and *Gs* could predict performance in the traditional L/T-letter search task and the X-ray image inspection task (SBST). Last, we tested whether the performance of the L/T-letter search task could mediate the effects of *Gv*, *Gms*, and *Gs* on the performance of the SBST.

### Descriptive Statistics and Correlations

Table 4 shows means and standard deviations of all independent (*Gs*, *Gv*, *Gsm*) and dependent variables (*d*_*a*_ SBST, RT SBST, *d*′ L/T, RT L/T) for students and professionals. Table 5 reports the Spearman correlations between all variables separately for students and professionals. Correlations with SPM scores served as a control and showed high significance with all the VCTB scales and a significant relationship with performance in both tasks. Correlations among the detection performance of the L/T-letter search task and SBST with the VCTB measures *Gv* and *Gsm* were all statistically significant within both populations. *Gs* correlated with detection performance of the L/T-letter search task for professionals and with the X-ray image inspection task for students. The intercorrelations of the VCTB scales were mostly in a medium range. We also correlated age as a control variable with both tasks as well as the VCTB scales. Within the population of professionals, we found negative correlations between age and SPM and between age and *Gs* as well as a positive correlation between age and detection performance in the SBST. These are expected results, because fluid intelligence, processing speed, and performance in SBST are known to decrease with age. In the student population, we did not find these relations. This could be due to the lower mean and range of age in this population.

**Table 4:** Means and standard deviations.

		**Students**				**Professionals**			
	**Max. score**	***n***	***M***	***SD***	**Cronbach's α**	***n***	***M***	***SD***	**Cronbach's α**
*d*_*a*_ SBST	3.5	128	1.6	0.3	0.83	112	2.6	0.4	0.80
RT SBST	4.0	128	3.2	1.1		112	2.6	0.7	
*d*′ L/T	3.5	128	1.0	0.5	0.71	112	1.0	0.5	0.70
RT L/T	15.0	128	8.1	1.3		112	8.2	11.4	
Gs	116	128	80.9	13.7	0.86–0.95	112	64.2	16.6	0.89–0.94
Gv	48	128	37.4	5.1	0.20–0.65	112	36.3	6.2	0.45–0.77
Gsm	31	128	21.7	5.5	0.56–0.75	112	19.8	5.7	0.62–0.68

*n, number of participants; M, mean; SD, standard deviation; Cronbach's α, internal consistency of scale; SBST, simulated baggage screening task; L/T-letter search task; Gs, processing speed; Gv, visual processing; Gsm, visual memory; statistical abbreviations: d_a_ and d′, detection performance measures; RT, reaction time in seconds*.

**Table 5:** Correlational analyses.

	**d_**a**_ SBST**	**RT SBST**	**d^**′**^ L/T**	**RT L/T**	**SPM**	**Gs**	**Gsm**	**Gv**
**STUDENTS**								
*d*_*a*_ SBST	–							
RT SBST	0.20^*^	–						
*d*′ L/T	0.34^***^	0.08	–					
RT L/T	0.23^**^	0.26^**^	0.45^***^	–				
SPM	0.28^**^	0.03	0.24^**^	0.20^*^	–			
Gs	0.22^*^	0.07	0.16	−0.03	0.57^***^	–		
Gsm	0.46^***^	0.23^**^	0.32^***^	0.25^**^	0.47^***^	0.33^***^	–	
Gv	0.40^***^	0.25^**^	0.35^***^	0.30^**^	0.37^***^	0.30^**^	0.64^***^	–
Age	0.19^*^	0.06	0.14	0.16	−0.03	−0.14	0.13	0.11
**PROFESSIONALS**								
*d*_*a*_ SBST	–							
RT SBST	0.18	–						
*d*′ L/T	0.35^***^	0.02	–					
RT L/T	0.23^*^	0.09	0.39^***^	–				
SPM	0.25^**^	−0.02	0.33^***^	0.21^*^	–			
Gs	0.11	−0.17	0.26^**^	0.02	0.61^***^	–		
Gsm	0.24^*^	0.07	0.28^**^	0.16	0.60^***^	0.43^***^	–	
Gv	0.39^***^	0.16	0.38^***^	0.34^***^	0.62^***^	0.43^***^	0.58^***^	–
Age	−0.05	0.48^***^	−0.03	−0.05	−0.19^*^	−0.36^***^	−0.15	−0.11

*Spearman Correlations*.

* *p < 0.05*.

** p < 0.01. and

*** *p < 0.001*.

### Measuring Model–Confirmatory Factor Analysis

In order to confirm the CHC-model structure of the VCTB scales, we constructed three latent factors: visual processing (*Gv*), short-term memory (*Gsm*), and perceptual speed (*Gs*). CFA showed that the theoretical model fitted the data well. All factor loadings reached statistical significance (*p* < 0.001), even though the factor loading of LPS10 was minimally under the recommended quality criterion of 0.50 (Hair et al., 2014) and the factor loading of LPS6 was clearly under 0.50. The overall model fit was good with Chi^2^ (32) = 56.56, *p* = 0.005, CFI = 0.961, TLI = 0.946 and RMSEA = 0.0359. As postulated by the CHC-model, the broad abilities of Stratum II were related, but distinct constructs. The correlation between the factors *Gs* and *Gsm* (*r* = 0.65, *p* < 0.001) as well as between *Gs* and *Gv* (*r* = 0.53, *p* < 0.001) was moderate, whereas there was a strong correlation between *Gsm* and *Gv* (*r* = 0.83, *p* < 0.001). The CHC-model structure was further tested for both populations separately and showed a good fit. This was taken as confirming the construct validity of the VCTB. For further analyses, we used the summarized and standardized scale scores of *Gv, Gsm*, and *Gs* in order to investigate those three abilities as more heterogeneous constructs.

### Multiple Linear Regression Analyses

In a next step, we calculated multiple linear regression analyses to predict detection performance on the L/T-letter search task and the SBST based on the *z*-standardized summarized scale scores of *Gv*, *Gsm*, and *Gs* and group (students vs. professionals). For predicting detection performance *d*′ on the L/T-letter search task, we found a significant regression equation *F*_(4, 235)_ = 9.64, *p* < 0.001, with an adjusted *R*^2^ of 0.13. *zGv* was the only significant predictor of detection performance (Table 6A). The same analysis was calculated again with group as moderator variable. However, the moderation did not improve the model fit (adjusted *R*^2^ = 0.12, see Table 6B) and the comparison of the two models using Wald's test did not reach statistical significance *F*_(3, 232)_ = 0.14, *p* = 0.939. Using the Bayes Factor to compare the two models revealed strong evidence against the moderation model (BF_10_ = 40.4).

**Table 6:** Multiple linear regression analyses and mediation model for detection performance.

	**L/T-letter search task (d^′^)**				**SBST (*d_a_*)**			
	**β**	***SE*(β)**	***t*-value**	***p*-value**	**β**	***SE*(β)**	***t*-value**	***p*-value**
**(A) BASIC MODEL**								
zGs	−0.013	0.078	−0.164	0.870	−0.039	0.044	−0.893	0.373
zGsm	0.119	0.079	1.513	0.132	0.104	0.044	2.348	0.019^*^
zGv	0.299	0.078	3.830	0.000^***^	0.195	0.044	4.463	0.000^***^
zGroup	0.029	0.070	−0.416	0.678	−0.834	0.039	−21.370	0.000^***^
**adj**. ***R**^**2**^*	0.126^***^				0.726^***^			
**(B) MODERATION MODEL**								
zGs	−0.018	0.079	−0.223	0.823	−0.03	0.044	−0.675	0.501
zGsm	0.127	0.080	1.567	0.119	0.113	0.045	2.533	0.012^*^
zGv	0.286	0.082	3.504	0.001^***^	0.190	0.045	4.132	0.000^***^
zGroup	−0.028	0.070	−0.400	0.700	−0.835	0.040	−21.458	0.000^***^
zGs*zGroup	−0.030	0.079	−0.378	0.705	0.064	0.044	1.461	0.145
zGsm*Group	0.036	0.080	0.451	0.652	0.064	0.045	1.426	0.155
zGv*Group	−0.034	0.080	−0.418	0.676	−0.054	0.045	−1.206	0.229
**adj**. ***R**^**2**^*	0.117^***^				0.730^***^			
					**β**	***SE*****(β)**	***t*****–value**	***p*****-value**
**(C) MEDIATION MODEL**								
zL/T d_a_					0.13	0.04	3.5	0.000^***^
zGs					−0.04	0.044	−0.88	0.382
zGsm					0.09	0.044	2.05	0.042^*^
zGv					0.16	0.044	3.58	0.000^***^
zGroup					−0.83	0.044	−21.77	0.000^***^
**adj**. ***R**^**2**^*					0.740^***^			

* p < 0.05; and

*** *p < 0.001*.

For predicting detection performance *d*_*a*_ on the SBST, we found a significant regression equation *F*_(4, 235)_ = 159.3, *p* < 0.001, with an adjusted *R*^2^ of 0.73. Group, *zGsm*, and *zGv* were significant predictors of detection performance (Table 6A). The same analysis was calculated again with group as moderator variable. However, the moderation did not improve the model fit (adjusted *R*^2^ = 0.73, see Table 6B) and the comparison of the two models using Wald's test did not reach statistical significance *F*_(3, 232)_ = 1.83, *p* = 0.143. Furthermore, we found strong evidence against the moderation model using the Bayes Factor (BF_10_ = 90.9). Because the explained variance was much higher in the SBST compared to the L/T-letter search task, we wanted to test whether this was due to the effect of group, which was only found for the SBST. When partialing out the group variable, the *R*^2^ decreased to 0.23. To further explore the effect of group, we tested whether work experience of professionals (years: *M* = 6.83, *SD* = 5.82) could explain some variance. However, there was no significant correlation between performance in the SBST and the log-transformed work experience (*p* = 0.09) and the model fit did not improve when including work experience as an additional variable (adjusted *R*^2^ = 0.72).

Up to this point, we found indication that both populations require the same visual-cognitive abilities to predict performance in both measured tasks. The regression models showed that performance on both visual search tasks was predicted by *zGv* and also *zGsm* (although only significantly for performance on SBST). Based on this result, it could be concluded that performance on, the L/T-letter search task and the SBST are predicted by the same visual-cognitive abilities. If this was the case, performance on the L/T-letter search task should fully mediate the effect of *zGv* and *zGsm* on performance in the SBST. This mediation effect would provide important information on whether results from traditional visual search tasks can be directly applied to professional X-ray image inspection. We investigated this hypothesis by conducting a mediation analysis using performance on the L/T-letter search task as mediator between the visual-cognitive abilities and performance on the SBST. We found a significant regression equation for the mediation model *F*_(5, 234)_ = 135.9, *p* < 0.001, with an adjusted *R*^2^ of 0.74. Table 6C shows that even though performance on the L/T-letter search task significantly predicted performance on the SBST, the direct effects of *Gv*, *Gsm*, and group still attained significance. The mediation model therefore showed that the effect of *Gv* and *Gsm* on performance of SBST was only partially mediated by performance on the L/T-letter search task. This means that L/T-letter search task performance by itself explains only part, but not all of the direct effects of *Gv* and *Gsm* on performance on the SBST, while *Gv* and *Gsm* explain an additional part of variance in performance on the SBST. To explore this result in more detail, we tested the size of the indirect effect of the visual-cognitive abilities on performance on the SBST through performance on the L/T-letter search task using bootstrapping procedures. These calculations give indication on how much variance of the total effect on performance on SBST can be explained by the effect of visual-cognitive abilities on performance on the L/T-letter search task, which in turn has an effect on performance on the SBST task. Indirect effects were computed for each of 10,000 bootstrapped samples, and the 95% confidence interval was computed by determining the indirect effects at the 2.5 and 97.5th percentiles. The bootstrapped indirect effects were 0.00 for *Gs* (*SD* = 0.01, 95% CI [−0.02, 0.02]); 0.01 for *Gsm* (*SD* = 0.01, 95% CI [−0.01, 0.04]); 0.04 for *Gv* (*SD* = 0.02, 95% CI [0.01, 0.08]); and −0.01 for group (*SD* = 0.02, 95% CI [−0.05, 0.03]). Thus, the indirect effects were small and not statistically significant, revealing that only a small part of the effect of *Gv* and *Gsm* on performance of the SBST was mediated by performance on the L/T-letter search task.

Since *Gs* did not show any effect on performance on the visual search tasks, we calculated the same analyses using response times (RT) as dependent variables (Table 7). For the L/T-letter search task, we found a significant regression equation *F*_(2, 235)_ = 10.95, *p* < 0.001, with an adjusted *R*^2^ of 0.14. *zGs* and *zGv* were significant predictors of response times (Table 7). We recalculated the same analysis including group as moderator variable. However, the moderation did not improve the model fit (adjusted *R*^2^ = 0.14) and the comparison of the two models using Wald's test did not reach statistical significance *F*_(3, 232)_ = 0.26, *p* = 0.85. Using the Bayes Factor for model comparison, results suggested strong evidence against the moderation model (BF_10_ = 37.46). For the SBST, the regression equation was also significant *F*_(4, 235)_ = 12.74, *p* < 0.001, with an adjusted *R*^2^ of 0.16. Group and *zGv* were significant predictors of response times (Table 7). Using group as moderator variable slightly improved the model fit (adjusted *R*^2^ = 0.18), however, the comparison of the two models using Wald's test did not reach statistical significance *F*_(3, 232)_ = 2.37, *p* = 0.07. Using the Bayes factor for model comparison, results suggested only weak evidence against the moderation model (BF_10_ = 2.40). Again, to further explore the effect of group, we entered work experience as an additional variable, but this did not improve the model fit (adjusted *R*^2^ = 0.18).

**Table 7:** Multiple linear regression analyses for response times (RT).

	**β**	***SE β***	***t-value***	***p*-value**
**L/T-LETTER SEARCH TASK**				
zGs	−0.209	0.077	−2.721	0.007^**^
zGsm	0.078	0.078	1.002	0.317
zGv	0.383	0.077	4.953	0.000^***^
Group	0.048	0.138	0.350	0.727
**X-RAY IMAGE INSPECTION TASK SBST**				
zGs	−0.149	0.076	−1.963	0.051
zGsm	0.114	0.077	1.484	0.139
zGv	0.176	0.076	2.307	0.022^*^
Group	−0.777	0.136	−5.699	0.000^***^

* *p < 0.05*;

** *p < 0.01*,

*** *p < 0.001*.

## Discussion

Many studies on the topic of visual search have been conducted with students using traditional, simplified visual search tasks and salient stimuli. Although such research is vital to explore the underlying cognitive mechanisms in a controlled environment, it is not always clear whether the results extrapolate to real-world inspection in which professionals search their visual fields for targets that are more complex, ambiguous, and less salient (e.g., Radvansky and Ashcraft, 2016, p. 257). Furthermore, visual search research is often conducted with students, who differ systematically from professional searchers. We investigated whether the same visual cognitive abilities predict performance in students and professionals performing two tasks: a traditional visual search task—the L/T-letter search task—and an X-ray image inspection task. We tested students and professionals on three known facets of visual-cognitive abilities: visual processing (*Gv*), short-term memory (*Gsm*), and processing speed (*Gs*). We shall now use our results to answer the following research questions: (1) Do different visual-cognitive abilities predict performance and response times in a traditional visual search task and an X-ray image inspection task? (2) Do the results differ between students and professionals?

Our results show that visual search ability as measured with a traditional visual search task involves different underlying visual-cognitive processes compared to an applied X-ray image inspection task. Whereas, visual search ability as measured with the L/T-letter search task was significantly predicted by visual processing (*Gv*), performance on the SBST was significantly predicted by visual processing (*Gv*) and short-term memory (*Gsm*). However, the mediation model revealed that only a small part of the effect of *Gv* and *Gsm* on performance of the SBST was mediated by performance on the L/T-letter search task. This leads to the conclusion that different aspects of *Gv* and *Gsm* predict performance in the measured tasks. Furthermore, the influence of the measured visual-cognitive abilities on performance did not differ between students and professional screeners. However, professionals outperformed students in the X-ray image inspection task.

### Traditional Visual Search vs. X-Ray Image Inspection

Multiple linear regression analyses were calculated for both visual search tasks in order to predict performance based on three visual-cognitive abilities (*Gv*, *Gsm*, *Gs*) and group (students vs. professionals). We further added the L/T-letter search task as a mediator of the effects of the visual-cognitive abilities to the model. The L/T-letter search task should reduce the direct effect of the visual-cognitive abilities on the X-ray image interpretation test if the two tasks depend on the same aspects of these abilities. However, the mediation model showed that only a small amount of the effects from the visual-cognitive abilities on X-ray image interpretation performance was mediated through the L/T-letter search performance. That different visual-cognitive abilities are relevant for the two tasks, is therefore indicated by the different underlying cognitive processes.

In the regression model, visual processing (*Gv*) was a predictor of performance for both tasks. This result is in accordance with earlier studies showing a correlation between performance and visual processing for traditional visual search (Wolfe et al., 2002; Bolfing and Schwaninger, 2009) and an influence of mental rotation and figure-ground segregation on higher performance in X-ray screening (Wolfe et al., 2002; Bolfing and Schwaninger, 2009), which are narrow abilities of visual processing (*Gv*). However, our results showed that different aspects of visual processing explain variance in the traditional visual search task and the X-ray image inspection task. According to the CHC theory, visual processing describes a broader ability to perceive, analyze, synthesize, and think with visual patterns, including the ability to store and recall visual representations. Both, the L/T-letter search task and the X-ray image inspection task require visual processing abilities, that is, the ability to mentally rotate objects and see them in their spatial relation and the ability to visualize and recognize patterns (e.g., visual memory, figure–ground segregation, or form constancy). However, visual processing includes a broad spectrum of abilities. Even though the traditional visual search task and X-ray image inspection task in this study were created to make them comparable, the tasks differed in regard to stimuli and distractor complexity. Targets in the traditional visual search task (*Ls* and *Ts*) have salient shapes, whereas targets (guns and knives) and distractors in the X-ray image inspection task are not salient and may additionally produce clutter and superposition. These are all potential reasons for our finding that different aspects of *Gv* are needed to perform faster and better in the measured tasks.

Short term memory (*Gsm*) was a significant predictor of X-ray image inspection performance, but not for the traditional visual search task. However, even though the standardized coefficient for *Gsm* was not smaller for the L/T-letter search task, it did not reach significance as a predictor for the L/T-letter search task (due to larger standard errors) and its relevance for that task is therefore unclear. *Gsm* is characterized as the ability to apprehend and hold information in immediate awareness and then use it within a few seconds. When comparing the stimulus complexity of the L/T-letter search task and the X-ray image inspection task, one would assume that *Gsm* might be especially important for a real-world task such as the SBST, which uses more complex and realistic stimuli and needs more top-down processing and the use of memory capacity, whereas simple letters are easy to remember. It can be further assumed that short-term memory becomes even more important when predicting performance in tasks with increasing complexity and unknown features that need previous knowledge. Regarding the X-ray image inspection task, the differentiation of targets from distractors needs memory capacity, because distractors appear in the form of everyday objects that can look similar to target items (Hättenschwiler et al., 2015; Sterchi et al., 2017), and prior object knowledge is needed to differentiate targets from non-targets.

Processing speed, the ability to quickly and accurately perceive visual details, similarities, and differences, did not predict detection performance in the measured tasks. We therefore additionally calculated a model for response times, in which processing speed predicted performance in the L/T-letter search task but fell short of significance for the X-ray image inspection task (significance in the SBST: *p* = 0.051). Participants with higher *Gs* scores therefore performed faster. This result is consistent with previous research that found processing speed to be relevant in terms of efficiency (Salthouse, 1996).

### Comparison of Students and Professionals

For both groups, visual-cognitive abilities were comparably relevant for their performance on the traditional visual search task and the X-ray image inspection task. However, professionals outperformed students on the X-ray image inspection task. Because the relevance of the visual-cognitive abilities tested in this study proved to be independent of the population and they had similar levels of visual-cognitive abilities, the higher detection performance of the professionals in the SBST cannot be explained by differences in visual-cognitive abilities. Consistent with this interpretation, after removing the group variable from the analyses in the X-ray image inspection task, a similar amount of variance could be explained as in the L/T-letter search task (especially when considering that the SBST was more reliable). This leaves mainly two possible explanations for this difference: Students and professionals might differ in other cognitive abilities than the ones measured, and these other abilities account for the improved detection performance only on the SBST but not the L/T-letter search task. Such a difference could be due to the selection of the security personnel. Or more likely, the group effect could be due to differences related to training and job experience of the professionals.

Halbherr et al. (2013) found that the biggest increase in performance is seen incrementally up to 40 h of training. The professionals participating in this study all had more than 2 years of training and work experience. Additional training hours might therefore not result in a large performance increase. This is consistent with our finding that partialling out age and work experience did not improve the model fit. McCarley et al. (2004) found detection performance improvements to be based on improvements in object recognition rather than the visual search task *per se*. Based on that, more familiar objects possibly need fewer recognized features in order to be identified successfully (Koller et al., 2009), and features are known and recognized better and faster with repeated exposure (McCarley et al., 2004; Schwaninger and Hofer, 2004; Koller et al., 2008, 2009; Halbherr et al., 2013). In our study, we created a traditional visual search task with a higher difficulty level and an X-ray image inspection task containing targets with no need of domain-specific knowledge. Features of guns and knives as well as letters such as *L* or *T* are known from everyday life and can therefore be detected without specific experience and training. However, the X-ray screening task requires the ability to resolve object occlusion, whereas the L/T-letter search task does not. Therefore, inferring the full shape of occluded objects may be superior in professionals due to higher object familiarity. It can further be assumed that work experience leads to richer object templates or representations of everyday objects in X-ray images (Hättenschwiler et al., 2015). As discussed above, distractors in an X-ray image inspection task are merely everyday objects that can look like threat items, especially if no target representation is stored. In comparison to a traditional L/T-letter search task in which distractors are salient and known, many everyday object distractors cannot be recognized easily in X-ray images without prior knowledge. This lack of knowledge can be a disadvantage for students who are not used to X-ray images and might lead them to incorrectly judge a bag to be harmful (Sterchi et al., 2017).

Regarding response times, the visual-cognitive abilities were comparably relevant for both groups in the traditional visual search task and the X-ray image inspection task. Using group as moderator variable only resulted in a small and not quite significant increase of the model fit. We, however, believe that this difference in *R*^2^ is too small to indicate a relevant moderation. Also the Bayes factor provides weak evidence against the moderation model. Therefore, differences between groups as discussed above only seem to be relevant for detection performance and not response times.

Taken together, the influence of the measured visual-cognitive abilities on performance did not differ between students and professional screeners. However, professionals outperformed students in the X-ray image inspection task, which we assume to be due to training and job experience of the professionals. The presence of a group difference, but apparent absence of a moderation suggests that experience (or any alternative reason for the group difference) does not interact with the relevance of the visual-cognitive abilities for the X-ray image inspection task. However, we would caution against assuming that this pattern can be generalized to other visual-cognitive abilities or other implementations of the X-ray image inspection task. The X-ray image inspection task as used in this study is not the same task as the one screeners conduct at checkpoints—particularly regarding target prevalence, coloring of images, and target categories. Prohibited items that are rather uncommon or have not been seen before (e.g., improvised explosive devices, IEDs) become very difficult to detect without training in the recognition of certain features of these threats (Schwaninger, 2004, 2005). Assuming that the performance in detecting such threats is still dependent on certain visual-cognitive abilities and that only professionals can detect them, these visual-cognitive abilities would only be relevant for the performance of professionals. We therefore expect that results would look different if a task was used that requires domain-specific knowledge.

## Limitations and Future Directions

One limitation of this study is the representativeness of the tested populations. Our samples of students and professionals showed similar means and standard deviations on the measured visual-cognitive abilities. Professionals participating in this study all passed a preemployment test for these visual abilities (e.g., X-Ray Object Recognition Test; see Hardmeier et al., 2005; Hardmeier and Schwaninger, 2008). It could therefore be possible that they have high levels of certain other relevant visual-cognitive abilites that were not included in this study. Future studies could investigate applicants for the screening job and investigate how far preemployment assessment limits variation in visual-cognitive abilities. It would further be interesting to observe whether the influence of the visual-cognitive abilities really remains stable when the screeners' performance increases through training and job experience. Further, the students tested in our study proved to be a very heterogeneous sample, especially with a high variance in age, which is not directly comparable to a typical student sample (students from universities of applied sciences tend to be more heterogeneous than students at other universities). This raises the question whether regression results would be affected if the tested sample were more homogeneous on some variables.

Our results suggest that different aspects of *Gv* and *Gsm* are relevant for performance on the L/T-letter search task and X-ray image inspection. Future studies should investigate the influence of narrow (Stratum I) abilities on these tasks. Implications based on current results could be that either a simple and short version of the visual-cognitive test battery (*Gv* scales) could be used to measure abilities and predict performance in students and professionals. Or in an applied setting, the SBST could be used as a criterion for abilities. Because there are major individual differences in visual-cognitive abilities, it should be tested whether someone is suited to perform well in a visual search and inspection task. Especially with regard to X-ray screening, airports could conduct preemployment assessments that test for certain visual abilities and aptitudes when recruiting new personnel. However, visual-cognitive abilities might become less important as performance predictors for tasks in which domain-specific knowledge is not only helpful but necessary. For example, when radiologists search for cancer in mammograms or screeners search for improvised explosive devices that include unknown features, training for these features should have a stronger influence on performance than visual-cognitive abilities. Future studies could also investigate whether visual-cognitive abilities change over time, and whether these abilities could be trained through repeated exposure to visual search tasks.

## Conclusion

With this study, we tried to determine how far results on a traditional visual search task can be translated to an X-ray image inspection and vice versa, and whether populations of students and professionals are comparable. Comparing visual-cognitive abilities and their influence on performance revealed that the different visual-cognitive abilities were able to predict performance on the measured tasks. The CHC proved to be a good model for mapping the visual-cognitive abilities needed to conduct a visual search task. Our mediation analyses revealed that the used tasks are not comparable *per se* as there was only a partial overlap between the required aspects of visual-cognitive abilities. Furthermore, although our tested populations were comparable in terms of performance predictors based on visual-cognitive abilities, professionals outperformed students on an applied X-ray image inspection task, suggesting that the performance is not solely predictable by visual-cognitive abilities. The implications of our second research question therefore have to be treated with caution, because the comparability of the two populations is dependent on the task. One should therefore be cautious about translating results from the L/T-letter search task to X-ray image inspection.

## Author Contributions

All authors substantially contributed to the conceptualization of the manuscript as well as to the acquisition, analysis, and interpretation of data. All authors critically revised the content of the manuscript repeatedly and approved the final version to be published. All authors agreed to be accountable for all aspects of the work. NH and SM as the leading authors contributed to the development of the tests, the acquisition, analysis, and interpretation of data. NH was responsible for the conceptualization and the writing of the manuscript. YS predominantly contributed to the acquisition, analyses, and interpretation of data. NH, SM, and YS repeatedly revised and refined the content of the manuscript critically. AS predominantly contributed to the development of the tests, the analyses and interpretation of data. AS repeatedly revised and refined the content of the manuscript critically.

### Conflict of Interest Statement

The authors declare that the research was conducted in the absence of any commercial or financial relationships that could be construed as a potential conflict of interest.

## Footnotes

^1^The choice between *d*_*a*_ and *d*′ would be a concern if there was systematic variance in the criterion. Although we did not expect this in our study, we recalculated the data using *d*′ and found no relevant differences in the results; that is, all significant effects remained significant.
